# Astaxanthin: A Compound in the Prevention of Chronic Diseases and as a Potential Adjuvant Treatment Agent

**DOI:** 10.3390/antiox14060715

**Published:** 2025-06-12

**Authors:** Xiao Zhu, Xi Chen, Matthew Wang, Honghua Hu

**Affiliations:** 1 School of Medicine, Jinhua University of Vocational Technology, Jinhua 321017, China; 2 Innovation Center of Translational Pharmacy, Jinhua Institute of Zhejiang University, Jinhua 321016, China; chenxi@zju-jhi.com; 3 Faculty of Medicine and Health, University of New South Wales, Sydney, NSW 2052, Australia; z5358422@unsw.edu.au; 4 College of Pharmaceutical Sciences, Zhejiang University, Hangzhou 310058, China; 5 Macquarie Medical School, Macquarie University, Sydney, NSW 2109, Australia

**Keywords:** astaxanthin, antioxidation, anti-inflammatory, anti-apoptosis, immune regulation, anti-tumor, chronic diseases

## Abstract

Astaxanthin (AST) is a fat-soluble carotenoid antioxidant. AST exhibits multiple protective mechanisms, including its antioxidant, anti-inflammatory, immunomodulatory, anti-apoptotic, nervous system-protective, anti-tumor, and anti-fibrotic effects. These effects make it a promising compound for the prevention of chronic diseases. AST can protect the nervous system against neurodegenerative diseases such as Alzheimer’s and Parkinson’s disease. It also protects the liver and helps reduce the risk of chronic kidney disease. Additionally, it improves cardiovascular health and has anti-diabetic properties. This review aims to provide an updated overview covering the protective effects of AST against various chronic diseases, including its antioxidant, anti-inflammatory, and anti-apoptotic effects. We also discuss the strategies used for improving astaxanthin bioavailability and its potential as an adjuvant therapeutic agent.

## 1. Introduction

### 1.1. Overview of Astaxanthin

Astaxanthin (AST) is a type of xanthophyll carotenoid, named 3,3′-dihydroxy-4,4′-diketo-β, β′-carotene, with the molecular formula C₄₀H₅₂O₄. It is a powerful antioxidant that widely exists in marine organisms (such as algae, crustaceans, and fish). *Haematococcus pluvialis* algae, in particular, has the highest natural AST content and is currently the most widely used source across various fields [1,2]. In the human diet, AST is found in marine animals such as salmon, trout, shrimp, and lobster. For instance, farmed Atlantic salmon contain 6–8 mg/kg of AST; European trout, 6 mg/kg; and Japanese trout, 25 mg/kg [3].

Its unique structure, featuring a cyclohexane ring with oxygen-containing β-ionone at both ends of the polyene chain enables AST to exhibit stronger antioxidant activity, being 14–65 times more effective than vitamin C, vitamin E, and β-carotene [4]. Animal and human studies indicate good AST tolerability with no significant toxicity. This may be due to ingested AST not being converted into vitamin A, preventing excess vitamin A accumulation from overconsumption [4,5]. In 1999, the U.S. Food and Drug Administration (FDA) approved AST as a dietary supplement. In 2010, the FDA granted “Generally Recognized As Safe (GRAS)” status to AST produced from *Haematococcus pluvialis*, the only current FDA-approved AST for direct human use [1]. There are three stereoisomers for AST: a pair of enantiomers (3S,3′S- and 3R,3′R-AST) and an optically inactivemeso form (3R,3′S AST [6] and geometric isomeric forms (Z and E isomers)) [6]. *Haematococcus pluvialis*-derived AST in the all-E-3S-3′ S form, is the most common type used as a dietary supplement and in clinical trials [3].

However, AST is highly susceptible to light, oxygen, and temperature and is easily degraded. Careful extraction and stabilization techniques are essential to preserve its integrity. Various methods can be employed to extract astaxanthin, including solvent extraction, ultrasound-assisted extraction, microwave-assisted extraction, supercritical fluid extraction, and enzymatic extraction [7]. Furthermore, encapsulation techniques are explored to enhance the stability and bioavailability of astaxanthin, demonstrating their effectiveness in protecting this valuable compound under challenging conditions [8].

### 1.2. The Health Benefits of Astaxanthin

**Figure 1:**
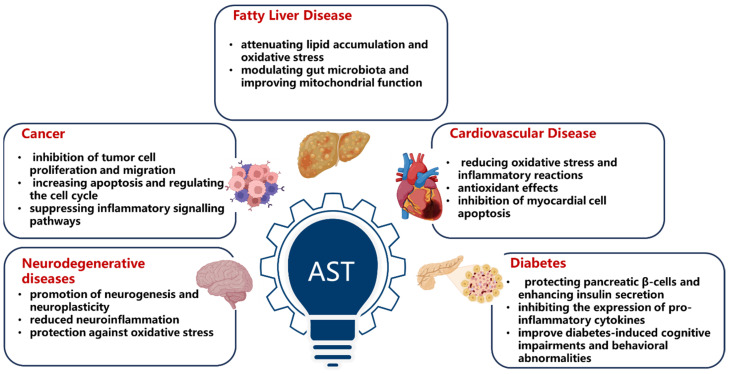
The beneficial effect of AST in preventing chronic diseases.

AST has gained recognition for its potential medicinal benefits in preventing and managing chronic diseases [9]. This compound offers a range of protective effects, including antioxidant, anti-inflammatory, anti-apoptotic, immunomodulatory, and anti-tumor properties. These effects make it effective in preventing and protecting against several chronic diseases, such as cardiovascular disease, diabetes, neurodegenerative disorders, liver diseases, and cancer, as illustrated in Figure 1.

These diseases are often associated with prolonged inflammation, oxidative stress, and cellular damage. Recent research indicates that AST may be a viable compound for modulating the pathophysiological processes associated with irreversible complications of disease progression. This offers a novel approach to enhancing quality of life through disease prevention and management.

### 1.3. Molecular Targets of Astaxanthin

The molecular targets of AST involve a variety of cellular signaling pathways, enzymes, and receptors, which are closely related to its antioxidant, anti-inflammatory, anti-apoptotic, and other biological activities and have a wide application potential in chronic disease prevention (Figure 1).

The main targets of AST include antioxidant systems; anti-inflammatory-, apoptosis-, and proliferation-related pathways; metabolism and signaling pathways; mitochondrial membrane protection, etc. The specific molecular targets and biological function of AST are summarized in Table 1.

## 2. Antioxidant and Anti-Inflammatory Effects and Mitochondrial Protection

### 2.1. Antioxidant Properties

AST is an antioxidant extensively studied for its ability to neutralize singlet oxygen, ROS known to cause cellular damage [17]. AST achieves its antioxidant effects primarily by directly scavenging free radicals and regulating the antioxidant enzyme system. The conjugated double-bond structure of astaxanthin allows it to directly capture free radicals, including superoxide anions (O_2_^−^), hydroxyl radicals (•OH), and peroxynitrite anions (ONOO^−^). This action helps block the chain reaction of lipid peroxidation. By reducing oxidative damage to cell membranes, DNA, and proteins, astaxanthin plays a protective role in cardiovascular and neurodegenerative diseases. AST has been known to provide cellular protection by activating the Nrf2-ARE signaling pathway, which promotes the upregulation of antioxidant enzymes such as SOD, GPX, and catalase [10], and enhances the endogenous antioxidant capacity of cells. Studies have shown that AST promotes the nuclear translocation of Nrf2 and increases the expression of its downstream proteins, heme oxygenase-1, and SOD1. AST also increased the activity of SOD and decreased malondialdehyde generation in the serum of diabetic rats. These results suggest that the protective effect of AST on diabetic nephropathy is partly dependent on Nrf2-ARE signaling [18]. This activation enhances the cell’s antioxidant defense, thereby reducing oxidative damage.

AST activates thioredoxin reductase (TrxR), maintains cellular redox balance, and inhibits cell apoptosis induced by oxidative stress. By mitigating oxidative stress—a key factor in many chronic diseases—AST enhances cellular resilience against oxidative damage caused by ROS [19].

### 2.2. Anti-Inflammatory Properties

AST alleviates chronic inflammatory responses by inhibiting inflammation-related molecular targets. Studies have found that AST can inhibit the positive feedback cycle of inflammatory factors by binding to IL-6, thus blocking the occurrence of inflammatory storm. RNA interference experiments further verified that IL-6 was the key target of its anti-inflammatory effect [1].

AST inhibits the activation of nuclear factor kappa-B (NF-κB) and its translocation to the nucleus, reducing the transcription of pro-inflammatory factors such as TNF-α, IL-1β, and IL-6, and plays an anti-inflammatory role in conditions like arthritis, hepatitis, and pulmonary inflammation. AST also inhibits the phosphorylation of the p38 MAPK pathway, JNK, and ERK, blocking the cascade amplification of inflammatory signals.

The anti-inflammatory actions of AST are closely linked to its antioxidant properties. These comprehensive antioxidant and anti-inflammatory effects suggest its potential for treating various diseases associated with oxidative stress and inflammation [20,21].

In conclusion, AST’s robust antioxidant and anti-inflammatory properties, coupled with its cellular protective mechanisms, position it as a valuable adjuvant therapeutic agent for mitigating diseases related to oxidative stress.

### 2.3. Mitochondrial Protection

AST can inhibit the lipid peroxidation of biological membranes and protect mitochondrial membranes against oxidative damage caused by ROS [20]. AST protects mitochondrial membrane from oxidative stress-induced damage [22]. AST added to cultured cells was transported to the mitochondria as most of the important components of the mitochondrial electric transport cells are located within the inner membrane of mitochondria [23]. AST also inhibits the opening of the mitochondrial permeability transition pore (mPTP) and reduces oxidative stress-induced cell damage and maintains mitochondrial function. For instance, AST has been shown to promote mitochondrial biogenesis and enhance energy metabolism in muscle cells through the AMPK/Sirtuins/PGC-1α pathway [15]. In vitro studies have demonstrated that AST can significantly reduce ROS generation, particularly in cells exposed to oxidative stressors such as hydrogen peroxide. For example, nanoparticles containing AST have been shown to decrease ROS levels in RAW 264.7 murine macrophage cell line. Mitochondrial-targeted AST nanoparticles exhibit an even more pronounced protective effect [22].

These findings suggest that astaxanthin’s ability to target mitochondria enhances its efficacy in reducing ROS production and protecting mitochondrial integrity. AST’s ability to modulate mitochondrial function further underscores its therapeutic versatility.

## 3. Immune Regulatory Effect

The regulatory effect on immune cells is crucial for controlling inflammation. For instance, a recent study demonstrated that AST suppresses oxidative stress and inflammatory factor production in LPS-induced dendritic cells via the HO-1/Nrf2 pathway [10], enhances defense mechanisms against pathogens, and reduces the incidence of autoimmune diseases. Recent studies have demonstrated that AST can modulate the immune system by inhibiting the maturation of immune cells, particularly dendritic cells, and by reducing the release of inflammatory factors [24]. By scavenging free radicals to reduce oxidative stress, AST helps maintain the integrity and function of SHP-1, a negative regulator of immune cytokine signaling crucial for dampening the inflammatory response. When SHP-1 is restored to its basal protein expression level, it can effectively inhibit the activation of the NF-κB signaling pathway. This inhibition prevents NF-κB from entering the nucleus and initiating the transcription of pro-inflammatory cytokines. Consequently, the reduced secretion of these pro-inflammatory cytokines, such as interleukin (IL-1β, IL-6) and tumor necrosis factor-alpha (TNF-α) mitigates the inflammatory response [25].

Extensive studies have elucidated the multifaceted mechanisms by which AST regulates the immune system, contributing to anti-infection, anti-inflammatory, and immunomodulatory effects, as summarized in Table 2.

A recent study demonstrated that AST significantly enhances both cellular and humoral immunity in murine models [26]. Specifically, AST promotes the proliferation and transformation of splenic lymphocytes, increases serum hemolysin levels, and enhances the activity of antibody-producing cells. Furthermore, AST significantly improves the carbon clearance rate in mice, indicating its capacity to strengthen nonspecific immune functions. These findings suggest that AST could fortify the body’s defense against pathogens by modulating the activity and function of immune cells. Li et al. [23] identified a novel mechanism by which AST enhances antiviral responses by inhibiting the carbonylation of STING (Stimulator of Interferon Genes). STING is an essential protein in the DNA-sensing pathway and crucial in antiviral immunity. Their study revealed that AST mitigated lipid peroxidation and inflammation induced by herpes simplex virus type 1 (HSV-1) while also augmenting type I interferon production, thereby restricting viral replication. This highlights the potential of AST to enhance antiviral defenses via modulating the STING signaling pathway. He et al. [27] explored the potential of AST to modulate immune responses in a mouse model of autoimmune hepatitis induced by Concanavalin A. They observed that AST significantly reduced liver damage and downregulated pro-inflammatory cytokines. Mass cytometry and single-cell RNA sequencing analyses revealed a substantial increase in CD8+ T cells in the liver following AST treatment, with downregulated expression of functional markers, such as CD69, MHC II, and PD-1. Specific CD8+ T cell subclusters (subclusters 4, 13, 24, and 27) exhibited distinct changes in marker gene expression, suggesting that AST may mitigate autoimmune hepatitis (AIH) by modulating the quantity and functionality of CD8+ T cells.

Despite the promising immunomodulatory effects of astaxanthin, inconsistencies across various studies highlight the complexity of its mechanisms. For instance, Nieman et al. [28] reported that while AST supplementation did not significantly reduce exercise-induced muscle soreness or damage, nor did it markedly affect plasma cytokine and oxylipin levels, it effectively countered the post-exercise decline in immune-related plasma proteins, particularly immunoglobulin IgM. These findings indicate that AST may play a role in modulating immune function under exercise-induced stress, although its effects may vary based on experimental design and environmental factors.

In summary, AST exhibits significant potential in modulating the immune system, enhancing defense mechanisms, and reducing the incidence of autoimmune diseases [29]. Various studies have elucidated its mechanisms, including the modulation of the STING signaling pathway [30], regulation of CD8+ T cell subclusters [27], and enhancement of immune cell activity [31]. However, the mechanisms characterizing AST’s effects may differ under diverse physiological and pathological conditions. Future research is warranted to clarify its specific roles in immune regulation and to explore its clinical applications.

**Table 2:** Immune regulatory effects of astaxanthin: evidence from animal studies.

Mechanism	Study Population/Model	Key Findings	Reference
Enhancement of both cellular and humoral immunity	SPF Kunming female mice	Significant improvement in delayed allergy reaction and NK cell activity	Fan et al. (2021) [26]
Inhibition in STING carbonylation to enhance antiviral responses	HSV-1-induced mouse primary peritoneal macrophages	Mitigating lipid peroxidation and inflammation, augments type I interferon production, restricting viral replication	Li et al. (2024) [32]
Downregulation of pro-inflammatory cytokines in autoimmune hepatitis	Concanavalin A-induced mouse model	Alleviating liver damage, downregulates pro-inflammatory cytokines, increases CD8+ T cells	He et al. (2024) [27]
Counteracting post-exercise decline in immune-related plasma proteins	Oplegnathus punctatus	Effective in counteracting the post-exercise decline in immune-related plasma proteins, particularly immunoglobulin IgM	Wu et al. (2023) [28]

## 4. Anti-Apoptotic Effect and Nervous System Protection

AST demonstrates anti-apoptotic effects by regulating apoptosis-related signaling pathways, which are particularly significant in the context of neurodegenerative diseases. As outlined in Table 3, AST shows potential neuroprotective effects against conditions such as Alzheimer’s disease (AD) and Parkinson’s disease (PD).

In AD, oxidative stress and mitochondrial dysfunction are key pathological mechanisms that contribute to neuronal apoptosis. Recent studies show that AST inhibits H_2_O_2_-induced excessive mitophagy and apoptosis by modulating the Akt/mTOR signaling pathway, thereby reducing oxidative stress-induced damage in neuronal cells [33,34,35]. This mechanism is crucial for protecting neurons from apoptosis and preserving cognitive function.

Animal studies have demonstrated that AST can enhance spatial memory performance by promoting neurogenesis and neuroplasticity. For example, AST has been shown to improve hippocampus-associated spatial memory by increasing the proliferation of neural progenitor cells and protecting them from oxidative damage [36]. Additionally, astaxanthin’s ability to upregulate brain-derived neurotrophic factors and activate the extracellular-signal-regulated kinase (ERK) pathway may further support synaptic plasticity and cognitive function [37]. In human studies, AST has been shown to reduce biomarkers associated with cognitive decline [38]. A randomized, double-blind, placebo-controlled study found that AST supplementation (6 mg/day or 12 mg/day) for 12 weeks significantly lowered phospholipid hydroperoxide concentrations in erythrocytes and plasma [39]. Elevated hydroperoxide levels have been associated with dementia, suggesting that AST may have preventative capabilities for Alzheimer’s disease. AST’s multifaceted neuroprotective effects, including its ability to inhibit amyloid-beta aggregation [40], reduce neuroinflammation [41], and protect against oxidative stress [42], position it as a valuable candidate for the prevention and adjuvant treatment of Alzheimer’s disease.

In PD, oxidative stress and neuroinflammation also contribute to dopaminergic neurons’ apoptosis [43]. AST can inhibit the activation of microglia and astrocytes, reducing the release of pro-inflammatory cytokines such as interleukins and tumor necrosis factors [44]. This anti-inflammatory action may help alleviate neuroinflammation and protect dopaminergic neurons. Astaxanthin’s neuroprotective effects in Parkinson’s disease may also be attributed to its modulation of signaling pathways involved in neuronal survival and function [45]. For instance, it can activate the PI3K/Akt pathway, which is crucial for neuronal protection and survival [46]. AST may enhance the expression of brain-derived neurotrophic factor, a key molecule for neuronal plasticity and repair [47]. While preclinical studies have shown promising results, clinical trials specifically investigating AST’s effects in PD patients are limited. However, the existing evidence suggests that AST could be a valuable adjunctive therapy for PD, potentially improving motor symptoms and cognitive function. Future research should focus on AST’s clinical trials to determine the optimal dosage, delivery methods, and long-term therapeutic efficacy in PD patients.

Its safety profile and ability to cross the blood–brain barrier further enhances its clinical potential [48]. In conclusion, astaxanthin’s role in reducing oxidative stress and regulating apoptosis signaling pathways offers a promising avenue for adjuvant treatment of neurodegenerative diseases. While direct evidence in AD and PD is still emerging, its mechanisms in other chronic diseases provide a strong theoretical basis for its neuroprotective potential. Future research should focus on understanding the specific role of AST in preventing these diseases and exploring its potential as an adjuvant therapeutic agent in clinical settings.

**Table 3:** Neuroprotective effects of astaxanthin: evidence from animal studies.

Mechanism	Study Population/Model	Key Findings	Reference
Improvement of brain aging	Six-month-old SAMP10 mice	Induction of autophagy by regulating IGF-1/Akt/mTOR and IGF-1/Akt/FoxO3a signaling pathways	Fu et al. (2023) [34,35]
Prevention of neurotoxicity	H_2_O_2_-induced SH-SY5Y cells	Inhibition H_2_O_2_-induced apoptosis in SH-SY5Y cells by ameliorating mitochondrial damage and enhancing cell survival	Yan et al. (2024) [34,35]
Promoting neurogenesis and neuroplasticity	Epidermal neural crest stem cells extracted from bulge hair follicle in adults	Enhances spatial memory performance, improves hippocampus-related spatial memory	Mohaghegh et al. (2020) [36]
Antioxidant properties	A mouse model of brain aging	Improvements in the learning, cognitive, and memory abilities of mice	Liu et al. (2021) [37]
Improvement of erythrocyte antioxidant status	Thirty middle-aged and senior subjects	Decreased PLOOH levels, which may contribute to the prevention of dementia	Nakagawa et al. (2011) [39]
Suppression of oxidative stress and apoptosis in neurons	PQ-induced SH-SY5Y cells and mice Parkinson’s disease model	Inhibition PQ-induced activation of MAPK signaling pathway	Wang et al. (2023) [44]
Anti-neuroapoptosis effects	Isofluorane-induced rat model	Reducing the isoflurane-induced neuroapoptosis via activation of the PI3K/Akt signaling pathway	Wang et al. (2016) [46]

## 5. Anti-Tumor Effect

AST possess significant anti-tumor potential by inhibiting cancer cell growth [49] and metastasis [50] and inducing tumor cell apoptosis as detailed in Table 4. AST blocks tumor progression by inhibiting the jak2/stat3 pathway and its downstream genes related to cell proliferation and angiogenesis [51,52]. It downregulates the expression of anti-apoptotic proteins (such as Bcl-2, p-BAD) and upregulates the expression of pro-apoptotic proteins to induce tumor cell apoptosis [13,53].

Notably, Shao et al. [54] demonstrated that AST suppresses the proliferation of prostate cancer DU145 cells by downregulating STAT3 expression, thereby enhancing apoptosis rates. Similarly, Faraone et al. [13] conducted a systematic review highlighting the anti-tumor activity of AST in colorectal cancer and melanoma, attributing its efficacy to the modulation of multiple molecular targets. This was further supported by Ni et al. [55], who demonstrated the inhibitory effects of AST on tumor growth in a PC-3 prostate cancer xenograft mouse model. Complementing these findings, Maoka et al. [56] elucidated the antioxidant properties of AST and its ability to scavenge peroxynitrite, thereby contributing to its anti-tumor and anticarcinogenic effects.

In the context of colorectal cancer, research indicates that AST significantly inhibits both tumor cell proliferation and migration. This response is largely attributed to its influence on key signaling pathways, namely the MAPK and NF-κB pathways, which reduce the expression of pro-inflammatory cytokines such as IL-6, IL-1β, and TNF-α [57]. As a result, AST helps mitigate oxidative stress and inflammatory responses, effectively slowing the progression of colorectal cancer. Additionally, astaxanthin’s ability to downregulate Ki67 expression further limits the malignant proliferation of tumor cells, underscoring its potential as a preventive and therapeutic agent for colorectal cancer [57]. Xu and Jiang [58] reported that AST exerts significant anti-tumor effects by inhibiting the proliferation, migration, and invasion of NPC C666-1 cells in nasopharyngeal carcinoma. These effects are achieved by suppressing PI3K/AKT and NF-κB signaling pathways, as evidenced by reduced levels of p-AKT, p-P65, and p-IκB. Additionally, AST upregulates miR-29a-3p expression, further inhibiting these signaling pathways, reinforcing its anti-tumor activity in NPC treatment. Furthermore, AST inhibits the proliferation and migration of esophageal cancer cells by upregulating PPARγ expression [59]. This effect is accompanied by the reduction in oxidative stress markers (e.g., MDA) and an increase in antioxidant enzyme activities (e.g., SOD and CAT). These findings collectively suggest that AST holds significant potential for preventing esophageal cancer. In glioblastoma multiforme, Shin et al. [60] observed a hormetic effect of astaxanthin, where low concentrations promoted cell proliferation while high concentrations induced apoptosis. This dose-dependent response highlights the complexity of astaxanthin’s role in GBM treatment. Furthermore, AST regulates the cell cycle by upregulating Cdk2 and p-Cdk2/3 expression while downregulating p53, thereby contributing to its anti-tumor effects.

These studies underscore the multifaceted anticancer potential of AST across various cancer types. Its mechanisms include inhibiting tumor cell proliferation [54] and migration [50], promoting apoptosis, regulating the cell cycle, suppressing inflammatory signaling pathways (e.g., MAPK and NF-κB) [57], and alleviating oxidative stress. Despite variations in its effects depending on cancer type, the consistent anti-tumor activity of AST is evident. Future research should focus on elucidating its safety and efficacy in clinical settings to establish AST as a viable therapeutic option for cancer patients.

## 6. Liver Protection

AST has gained significant attention for its potential protective effects on the liver, particularly in cases of metabolic dysfunction-associated steatotic liver disease (MASLD) and alcoholic liver disease (ALD). Evidence shows that AST mitigates liver injury through its anti-inflammatory actions by inhibiting NF-κB activation, antioxidant effects by upregulating glutathione levels, and metabolic regulatory actions by modulating lipid metabolism pathways. A key focus is its ability to upregulate fibroblast growth factor 21 (FGF21) and peroxisome proliferator-activated receptor gamma coactivator 1α (PGC-1α).

In MASLD, AST shows strong protective effects by reducing lipid accumulation and oxidative stress. For example, Wu et al. [61] demonstrated that AST significantly decreased hepatic lipid deposition in models of MASLD induced by a high-fat diet. This improvement was accompanied by enhanced mitochondrial function, attributed to the upregulation of FGF21 and PGC-1α. These findings highlight astaxanthin’s role in improving mitochondrial biogenesis and oxidative phosphorylation, which can aid in slowing MASLD progression. Additionally, AST regulates the expression of critical genes involved in lipid metabolism, inhibiting the uptake and synthesis of fatty acids while promoting their oxidation, collectively leading to reduced hepatic lipid accumulation.

In the case of ALD, AST alleviates alcohol-induced liver injury by modulating gut microbiota and improving mitochondrial function. Liu et al. [62] demonstrated that AST significantly altered the gut microbiota composition, reducing the abundance of pro-inflammatory bacteria while increasing beneficial species such as *Akkermansia muciniphila*. This shift in microbiota composition was associated with improved gut barrier function, reduced systemic inflammation, and decreased alcohol-induced liver damage. Furthermore, Krestinina et al. [63] clarified astaxanthin’s protective effects against alcohol-induced mitochondrial dysfunction. Their study demonstrated that AST restored mitochondrial respiratory function and oxidative phosphorylation activity by upregulating the expression of mitochondrial respiratory chain complexes, thereby reducing alcohol-induced hepatic injury.

Overall, these studies revealed the various protective mechanisms of AST in both MASLD and ALD. It alleviates liver injury by decreasing oxidative stress and inflammation, enhancing mitochondrial function, upregulating FGF21 and PGC-1α [61], and modulating gut microbiota to improve gut barrier integrity [62]. These findings position AST as a promising therapeutic candidate for managing MASLD and ALD. Future research should clarify the detailed molecular pathways involved in astaxanthin’s protective effects on the liver and explore its clinical potential in human studies.

## 7. Anti-Fibrotic Effect

Astaxanthin, a carotenoid, has gained significant attention due to its powerful antioxidant, anti-inflammatory, and immunomodulatory properties. In particular, its potential for addressing fibrosis-related diseases has been increasingly recognized. AST inhibits renal fibrosis by modulating the Smad2, Akt, and STAT3 signaling pathways and pulmonary fibrosis by regulating long non-coding RNA and mitochondrial signaling pathways. These effects primarily regulate specific signaling pathways that suppress fibroblast activation and epithelial–mesenchymal transition (EMT).

Studies have shown that AST can exert anti-fibrotic effects through multiple pathways. For instance, Diao et al. [64] utilized a unilateral ureteral obstruction mouse model and found that AST significantly alleviated renal fibrosis. The mechanisms involved inhibiting fibroblast activation by modulating the Smad2, Akt, and STAT3 signaling pathways, also suppressing EMT in renal tubular epithelial cells through the Smad2, Snail, and β-catenin pathways. AST promotes the accumulation of CD8+ T cells in the kidneys by upregulating the expression of CCL5, thereby inhibiting renal fibrosis. Another study further revealed that AST mitigates renal fibrosis and peritubular capillary rarefaction by inhibiting the activation of the TGF-β1/Smad signaling pathway. These findings suggest that AST holds therapeutic potential in treating renal fibrosis [65].

Chen et al. [66] demonstrated that AST can alleviate pulmonary fibrosis by inhibiting the proliferation and migration of activated fibroblasts through long non-coding RNA (lncITPF) and mitochondria-mediated signaling pathways. Specifically, AST suppresses the expression of lncITPF by inhibiting the phosphorylation and nuclear translocation of Smad3, thereby reducing the expression of its target gene ITGBL1. Moreover, AST promotes the apoptosis of activated fibroblasts by regulating Drp1-mediated mitochondrial fission. These discoveries elucidate the anti-fibrotic mechanisms of AST in pulmonary fibrosis, providing a theoretical foundation for developing new therapeutic strategies.

Although these studies have revealed the anti-fibrotic effects of AST in different fibrosis models, some differences exist. For instance, in renal fibrosis, AST mainly exerts its effects by modulating cellular signaling pathways and immune cell infiltration, while in pulmonary fibrosis, its mechanisms primarily focus on regulating fibroblast behavior through lncRNA and mitochondrial signaling pathways. Additionally, despite the demonstrated anti-fibrotic potential of AST in various fibrosis models, its mechanisms of action may exhibit tissue-specific characteristics, which require further investigation.

As an anti-fibrotic compound, AST inhibits fibroblast activation and the EMT process through multiple mechanisms and shows promising therapeutic prospects in treating renal and pulmonary fibrosis. Future research is needed to explore the mechanisms of action in different fibrotic diseases and evaluate the feasibility and safety of its clinical application.

## 8. Cardiovascular Health Improvement

AST has gained attention for its potential role in preventing and treating cardiovascular diseases (CVD). AST’s cardiovascular protective effects have been observed in cellular models, animal studies, and human trials, emphasizing its potential application in CVD treatment (Table 5).

The antioxidant activity of AST has been extensively studied in various CVD-related cell culture models, including endothelial cells, macrophages, and T cells [2]. For instance, in cultures of human umbilical vein endothelial cells (HUVECs), the addition of AST significantly reduces ROS-induced lipid peroxidation and enhances the activity of antioxidant enzymes [67]. Speranza et al. [25] found that treating human macrophage-like U397 cells with 10 μmol/L of AST for 24 h could alleviate hydrogen peroxide-induced cytotoxicity, while AST itself showed no toxicity to these cells. Subsequent experiments from the same research group revealed that U397 cells pre-treated with AST (10 μmol/L) for 1 h reduced lipopolysaccharide-induced superoxide production and maintained the activity of intracellular antioxidant enzymes [68].

AST has a protective effect on CVD such as atherosclerosis by neutralizing ROS/RNS) and regulating inflammatory pathways (such as NF-κB) [69]. In addition to directly lowering ROS to exert antioxidant effects, AST can also act as an antioxidant by regulating related oxidases and oxidative-stress-related signaling pathways. Recent years have seen growing interest in the antioxidant activity of paraoxonase 1 (PON1) and its relationship with CVD [70,71]. Studies indicated that PON1 can protect lipoproteins from oxidative modification, and observations indicated that individuals with low PON1 levels are at an increased risk of CVD. Current in vitro experimental results suggest that a high concentration of synthetic AST (20 μmol/L) can enhance cellular antioxidant capacity by inducing PON1 [72]. Similarly, when AST (50–500 mg/100 g feed) was added to the feed of hypercholesterolemic rabbits, it prevented a decline in PON1 activity [73]. In trials with healthy subjects who were administered AST, there was an increase in PON1 activity, suggesting that AST may exert its effects by modulating the activity of this enzyme [74].

A recent review highlighted the significance of the Nrf2-ARE signaling pathway in CVD [75,76]. However, the ability of AST to activate the Nrf2-ARE signaling pathway in cellular experiments remains controversial, and if it exists, the activation capacity is relatively weak [68,72,77,78]. In animal studies, the oral administration of AST at 25 mg/kg body weight (bw) to rats increased the expression of Nrf2 and its downstream target HO-1 [79].

Chronic inflammation is a hallmark of atherosclerosis, with various immune mediators (such as chemokines and lipids) and inflammatory signaling pathways (including MAPK, PI3K, JAK/STAT, and NF-κB) identified as targets for CVD [80,81]. In vitro, AST has been shown to reduce multiple inflammatory markers within various cells, such as rat alveolar macrophages, U937 human cell lines, THP-1 cells, and HUVECs [82,83]. In all studies, the anti-inflammatory activity of AST is primarily attributed to its role in reducing reactive free radicals. Various animal studies conducted on mice, rats, chickens, and dogs have provided evidence that AST can reduce the inflammatory burden [84,85,86,87]. For instance, Chan et al. [85] added 0.01% or 0.05% AST to the feed of diabetic rats and found that after 12 weeks, the levels of IL-6, TNF-α, monocyte chemoattractant protein-1 (MCP-1), and von Willebrand factor in the rats’ serum significantly decreased.

AST has demonstrated reproducible and significant antihypertensive effects in rat models. Hussein et al. [88] found that after 14 days, oral administration of AST reduced blood pressure in spontaneously hypertensive rats, while Wistar Kyoto rats remained unchanged. After 18 weeks of supplementation with 50 mg/kg AST in metabolic syndrome rats, blood pressure significantly dropped [89]. Other studies also reported reductions in systolic blood pressure after 8 weeks of AST administration in SHR rats [65]. However, Preusse et al. [90] found that only the 100 mg/kg AST group had significant blood pressure reduction after 8 months. The antihypertensive mechanisms of AST likely involve nitric oxide and oxidative stress, possibly enhancing nitric oxide production and inducing vasodilation [90,91].

AST has also demonstrated hypolipidemic effects. AST downregulates the expression of lipogenesis genes (such as Glycerol-3-phosphate dehydrogenase) and reduces triglyceride accumulation in 3T3-L1 adipocytes [92]. A study conducted by Galema-Boers et al. indicates that elevated levels of total cholesterol (TC), low-density lipoprotein cholesterol (LDL-C), and triglycerides (TGs), along with reduced levels of high-density lipoprotein cholesterol (HDL-C), can lead to dyslipidemia and increase the risk of CVD [93].

In animal studies, dietary AST significantly lowered plasma triglyceride levels and increased HDL-C in metabolic syndrome models [94,95]. AST also reduced adipocyte size by increasing adiponectin levels. AST-treated mice showed enhanced expression of liver cholesterol metabolism genes (LDL receptor, 3-hydroxy-3-methylglutaryl-CoA reductase, and sterol regulatory element-binding protein) and β-oxidation-related genes (carnitine palmitoyltransferase-1 and acyl-CoA), but no significant changes in lipogenic gene expression [96].

The disruption of atherosclerotic plaques and subsequent thrombotic responses to vascular injury are critical contributors to clinical cardiovascular events, including myocardial infarction and stroke. Sasaki et al. [97] demonstrated that in spontaneously hypertensive rats with a high risk of stroke, supplementation with AST or vitamin E reduced systolic blood pressure, delayed cerebral vascular thrombosis, increased the nitric oxide (NO) metabolic rate, elevated urinary NO₂/NO₃ levels, and significantly decreased 8-OHdG levels. Complementing these findings, Khan et al. [98] observed that C57BL/6 mice treated with CDX-085 exhibited significantly increased basilar artery blood flow and a delayed thrombosis following endothelial injury. In vitro experiments by the same group further revealed that AST could substantially enhance NO levels, reduce ONOO^−^ levels, promote vasodilation in the aortic and coronary arteries, increase blood flow, decrease blood viscosity, protect LDL from oxidation, and inhibit primary thrombus formation.

Human trials reviewed by Fasset et al. [4] suggested AST reduces lipid oxidation in healthy individuals and lowers oxidative stress in those at risk for atherosclerosis. A randomized trial demonstrated that AST intake reduced blood lipid peroxidation markers and increased antioxidant levels in overweight adults [99]. Young women supplementing with AST showed improved inflammation markers and immune responses and enhanced cytokine profiles, with the low-dose group (2 mg/d) showing better effects compared to the high-dose group (8 mg/d) [100]. However, natural AST did not significantly affect plasma C-reactive protein levels in renal transplant patients [101].

In summary, AST exerts protective effects on the cardiovascular system through multiple mechanisms, including antioxidant activity, anti-inflammatory effects, apoptosis inhibition, and modulation of cellular signaling pathways. These findings highlight the potential therapeutic value of AST in cardiovascular diseases and provide a theoretical foundation for future clinical research and drug development. Nonetheless, larger-scale and long-term clinical trials are still needed to validate the efficacy and safety of AST in cardiovascular disease treatment.

**Table 5:** Cardiovascular health improvement of astaxanthin: evidence from animal studies.

Mechanism	Study Population/Model	Key Findings	Reference
Antioxidant Properties	Human umbilical vein endothelial cell	Reducing ROS-induced lipid peroxidation and enhancing antioxidant enzyme activity	Nishigaki et al. (2010) [67]
	U397 cell	Restoring SHP-1 expression and reducing NF-κB (p65) nuclear expression	Speranza et al. (2012) [25]
	U397 cell	Reducing LPS-induced toxicity and ROS production by decreasing intracellular O₂ (−) production	Franceschelli et al. (2014) [68]
	HepG2 cell	Enhancing cellular antioxidant capacity by inducing PON1	Dose et al. (2016) [72]
	Hypercholesterolemic rabbits	Regulator of PON1 activity	Augusti et al. (2012) [73]
	Healthy subjects	Increasing PON1 activity	Baralic et al. (2013) [74]
	Rats	Increasing the expression of Nrf2 and its downstream target HO-1	Tripathi et al. (2009) [79]
Anti-inflammatory Effects	Diabetic rats	Reducing multiple inflammatory markers	Chan et al. (2012) [85]
Antihypertensive Effects	Spontaneously hypertensive rats	Modulating blood fluidity and improving vascular reactivity	Hussein et al. (2005) [88]
	Metabolic syndrome rats (SHR/NDmcr-cp model)	Inducing a significant reduction in arterial blood pressure in metabolic syndrome rats	Hussein et al. (2007) [89]
	Male Sprague-Dawley rats	Enhancing nitric oxide, thereby inducing vasodilation of the rat aorta	Preuss et al. (2011) [90]
	Spontaneously hypertensive rats	Lowering the ratio of coronary artery vessel wall to lumen, reducing the increase in aortic elastin	Hussein et al. (2006) [91]
Hypolipidemic Effects	Male C57BL/6J mice fed a high-fat diet	Lowering the plasma concentrations of TAG, ALT, and AST	Yang et al. (2014) [94]
	Metabolic syndrome rats	Significantly increasing HDL-C levels and decreasing plasma TG and non-esterified fatty acid levels in a metabolic syndrome rat model	Yoshida et al. (2010) [95]
	APOE-knockout mice fed a high-fat, high-cholesterol diet	Increasing expression of liver cholesterol metabolism-related genes and major β-oxidation-related enzymes	Yang et al. (2011) [96]
Antithrombotic Effects	Spontaneously hypertensive rats	Reduction in systolic blood pressure, a delay in cerebral vascular thrombosis, an increase in NO metabolic rate, a significant elevation in urinary NO₂/NO₃ levels, and a marked decrease in 8-OHdG levels	Sasaki et al. (2011) [97]
	C57BL/6 mice	Substantially enhancing NO levels, reducing ONOO^−^ levels, promoting vasodilation in the aortic and coronary arteries, augmenting blood flow, decreasing blood viscosity, and protecting LDL from oxidation	Khan et al. (2010) [98]
Cardioprotective Effects	Healthy subjects	Reducing the oxidation of fatty acids and LDL	Fassett et al. (2012) [4]
	Overweight and obese adults	Decreasing lipid peroxidation markers in the blood significantly and increasing plasma superoxide dismutase levels and total antioxidant levels	Choi et al. (2011) [99]
	Young, healthy women	Lower levels of C-reactive protein (an inflammatory marker used to determine CVD risk), improving immune cell responses and cytokine status	Park et al. (2010) [100]
	Renal transplant patients	Having no significant effect on plasma C-reactive protein levels	Coombes et al. (2016) [101]

## 9. Anti-Diabetes Effect

AST exerts therapeutic effects on diabetes and its complications through antioxidant actions, which protect pancreatic β-cells, and anti-inflammatory effects, which reduce insulin resistance. Additionally, it can alleviate diabetes-induced behavioral abnormalities by reducing oxidative stress and neuroinflammation in the brain (Table 6).

Hyperglycemia in diabetes triggers excessive production of ROS, leading to pancreatic β-cell dysfunction, insulin resistance, and endothelial cell damage. AST has been shown to effectively scavenge ROS and reduce oxidative stress, thereby protecting pancreatic β-cells and enhancing insulin secretion [102]. It also regulates the expression of antioxidant-related genes and strengthens the endogenous antioxidant system, further mitigating cell oxidative damage [103]. These antioxidant effects are crucial for alleviating the oxidative burden associated with diabetes.

Chronic low-grade inflammation is a hallmark of diabetes and its complications. AST has demonstrated significant anti-inflammatory effects by inhibiting the expression of pro-inflammatory cytokines such as TNF-α, IL-6, and MCP-1, thereby reducing inflammation [104]. This anti-inflammatory activity improves insulin resistance, and also slows the progression of diabetic complications, including nephropathy, retinopathy, and neuropathy [105].

Diabetes is frequently associated with behavioral abnormalities, including cognitive decline, anxiety, and depression, conditions often linked to oxidative stress and neuroinflammation. Studies have shown that AST can improve diabetes-induced cognitive impairments and behavioral abnormalities by attenuating oxidative stress and neuroinflammation [41,42,106]. For instance, in diabetic rat models, AST significantly enhanced cognitive function by activating the PI3K/Akt signaling pathway, thereby reducing oxidative stress and neuroinflammation [106].

AST activates PPARs, PPARγ, and PPARα, which regulate lipid metabolism and glucose homeostasis and improve insulin resistance [59].

## 10. Strategies for Improving Astaxanthin Bioavailability

AST is known for its strong antioxidant activity; however, its clinical application is limited due to low oral bioavailability, primarily caused by its high lipophilicity and low water solubility. Furthermore, the various isomer forms of AST also influence its bioavailability.

Several strategies can be employed to enhance AST’s bioavailability (see Table 7), including novel delivery systems, such as lipid-based carriers, nano delivery with a sustained-release (SR) system, and targeted delivery systems, and structural modifications, such as esterification, isomer form selection, etc.

Dietary fat enhances small-intestine absorption of AST, while smoking reduces its elimination half-life [107]. Co-consuming AST with fat can greatly increase its bioavailability. Research shows that the area under the curve (AUC) for the serum concentration of AST is significantly higher when taken after meals than on an empty stomach [107].

Using lipid carriers, such as microemulsions and liposomes, can greatly enhance AST’s solubility and absorption. For instance, AST liposomes coated with soybean lecithin show improved biological accessibility during simulated digestion [108]. Additionally, lipid-based preparations containing AST have been shown to provide superior absorption compared to regular food supplements [109].

Nanoemulsions, solid lipid nanoparticles, chitosan, or poly lactic-co-glycolic acid (PLGA) nanoparticles can enhance the stability and targeted delivery of AST [110,111]. AST was encapsulated in PLGA nanoparticles coated with chitosan oligosaccharides, having good dispersibility and stability in aqueous solutions, as well as high cytocompatibility [110]. PLGA nanoparticles, in particular, can provide controlled release and improve bioavailability [112]. The bioavailability of AST with a SR matrix formulation is 3.6 times higher than that of AST oil without formulation [113].

Microencapsulation technologies, such as spray drying, can protect AST from degradation and enhance its release characteristics [114]. Whey protein microcapsules exhibiting microencapsulation have demonstrated a bioavailability that is 3.15 times higher than that of a control group [114]. Microencapsulated AST, prepared using soybean lecithin through the spray drying method, can improve both water solubility and absorption [114,115].

The molecular structure, particularly the isomer forms, also affects bioavailability and absorption efficiency. Different optical stereoisomeric forms (3S-3′S, 3R-3′S, 3R-3′R) and geometric isomeric forms (Z and E isomers) demonstrate varying effects [6]. Studies indicate that the plasma concentration of 9z- and 13z-AST is higher than that of all-E AST, possibly due to better transport efficiency [116].

The degree of esterification (mono-ester or di-ester forms) also impacts AST’s bioavailability. For example, AST esters, such as diethyl ASTA and ASTA es, are more stable and have higher bioavailability than free AST [117]. Furthermore, short-chain fatty acid esters exhibit better absorption efficiency than long-chain esters [117].

AST polyethylene glycol succinate (APGS) has shown improved solubility, bioavailability, and stability compared to free AST [102]. In tests conducted on type 2 diabetic mice, hydrophilic APGS was found to have higher bioavailability and a more favorable effect on diabetes and inflammation, attributing this to its favorable pharmacokinetic behavior and water solubility [102]. Polyethylene glycol (PEG)-carotenoid ester conjugates have also demonstrated significant antioxidant activity due to their water dispersibility [118].

Other formulations and advanced technologies aimed at improving AST bioavailability include synthetic AST, micellar solubilization technology, and targeted delivery systems. For example, Cardax, a novel synthetic AST derivative, displays enhanced water solubility and bioavailability compared to free AST [119]. In healthy individuals, Novasol capsules, based on micellar solubilization technology, have shown higher plasma concentrations and improved pharmacokinetic parameters [120]. Additionally, research conducted by Liu et al. on an intestine-targeted delivery system demonstrated remarkable mucoadhesive capacity, efficient deep mucus layer penetration, and enhanced absorption of AST to improve its bioavailability in vivo [121].

In conclusion, leveraging novel delivery systems, such as lipid-based carriers, nano delivery mechanisms, sustained-release formulations, and targeted delivery systems, alongside structural modifications like esterification and optimizing isomer forms, can significantly enhance the bioavailability of AST.

**Table 7:** Strategies for improving AST bioavailability.

Strategy	Specific Measures	Results	Reference
Lipid-based Carriers	Co-intake of AST with dietary fat	AUC of the serum concentration of AST taken after meals was significantly higher than that taken on an empty stomach	Okada et al. (2009) [107]
	Using lipid carriers like microemulsions and liposomes	AST liposomes coated with soybean lecithin significantly improved their biological accessibility in simulated digestion	Chang et al. (2022) [108]
		Three lipid-based formulations of AST all showed enhanced bioavailability, ranging from 1.7 to 3.7 times that of the reference formulation	Mercke et al. (2003) [109]
Nano Delivery and SR System	Using nanoemulsions, solid lipid nanoparticles, chitosan or PLGA nanoparticles	AST was encapsulated in PLGA nanoparticles coated with chitosan oligosaccharides, with good dispersibility and stability in aqueous solutions, as well as high cytocompatibility	Liu et al. (2019) [110]
		PLGA nanoparticles can achieve sustained release and improve bioavailability of AST	Xue et al. (2023) [112]
	SR formulation	The bioavailability of AST with an SR matrix formulation is 3.6 times higher than that of AST oil without formulation	Madhavi et al. (2018) [113]
Microencapsulation	Microencapsulation technology like spray drying (e.g., whey protein microcapsules, soybean lecithin microcapsules)	WP encapsulation can effectively improve the stability, water solubility, and bioavailability of AST esters	Yang et al. (2022) [114]
		AST was microencapsulated with soluble polymers using spray drying to improve its solubility and bioavailability	Nalawade et al. (2015) [115]
Isomer Optimization	Studying molecular structures (optical stereoisomers and geometric isomers)	13Z-AST showed higher bioaccessibility than 9Z- and all-E-ASTs during in vitro digestion, and 9Z-AST exhibited higher transport efficiency than all-E- and 13Z-ASTs	Yang et al. (2016) [116]
	Using *Haematococcus pluvialis*-derived AST (all-E-3S-3′ S form)	A selective process increases the relative proportion of AST Z-isomers compared to the all-E-AST before uptake in blood and that the AST esters are hydrolyzed selectively during absorption	Coral-Hinostroza et al. (2004) [122]
		A selective process increases the relative proportion of AST Z-isomers compared to the all-E-AST during blood uptake and that AST E/Z isomers have similar pharmacokinetics	Østerlie et al. (2000) [123]
Esterified Form	Using esterified AST	AST ester (such as diethyl ASTA de, ASTA es) is more stable and has higher bioavailability than free AST	Yang et al. (2020) [117]
		AST polyethylene glycol succinate (APGS) showed better solubility with enhanced bioavailability and stability compared to free AST	Sakayanathan et al. (2024) [102]
		PEG−carotenoid ester conjugates also showed good antioxidant activity due to their water dispersibility	Háda et al. (2011) [118]
Other Formulations and Advances	Synthetic AST	Cardax, a novel synthetic AST disodium disuccinate derivative, exhibits higher water solubility and bioavailability than free AST	Lockwood et al. (2005) [119]
	Micellar solubilization technology	In healthy men, the Novasol capsule, based on micellar solubilization technology, showed higher plasma concentration and better pharmacokinetic parameters	Khayyal et al. (2024) [120]
	Intestine-targeted delivery systems	A gut-responsive carrier remarkably increased muco-adhesion, deep mucus layer penetration, and AST absorption and bioavailability	Liu et al. (2024) [121]

## 11. Discussion and Conclusions

AST has gained significant attention for its potential as a therapeutic agent against various chronic diseases. This review summarizes the mechanisms behind AST’s antioxidant, anti-inflammatory, anti-apoptotic, immunomodulatory, anti-tumor, and anti-fibrotic activities, as well as strategies to enhance its bioavailability. These diverse functions position AST as a promising compound for managing various chronic conditions.

AST’s ability to ROS and modulate gene expression related to oxidative stress offers neuroprotective effects against neurodegenerative diseases such as AD and PD. Moreover, AST’s capacity to inhibit pro-inflammatory cytokines and regulate inflammatory signaling pathways protects against chronic inflammatory conditions, including chronic obstructive pulmonary disease (COPD), cardiovascular diseases, liver and chronic kidney disease, while also providing anti-diabetic benefits.

Despite these promising findings, several limitations and challenges remain in current research. The precise mechanisms of action for AST have not yet been fully clarified, particularly concerning its specific targets and signaling pathways in various pathological conditions. Additionally, the relatively low bioavailability of AST may limit its absorption and distribution in the body, which ultimately affects its therapeutic efficacy [103]. Novel delivery systems and structural modifications have shown promise in improving AST’s bioavailability and enhancing therapeutic outcomes.

Future research should focus on elucidating the molecular mechanisms of AST, exploring its pharmacokinetics, and developing more cost-effective industrial production methods. First, a deeper investigation into AST’s molecular mechanisms in various chronic diseases is needed, especially regarding its targets and signaling pathways in neurodegenerative and metabolic disorders. Second, more clinical trials are necessary to confirm the efficacy and safety of AST in humans and to determine optimal dosing and administration protocols. Lastly, considering AST’s multi-target nature, future studies should investigate its potential in combination therapies to achieve synergistic effects.

In conclusion, AST shows considerable promise as a compound for preventing various chronic diseases and may serve as an adjuvant treatment for chronic conditions.

## Figures and Tables

**Table 1:** Molecular targets and biological function of astaxanthin.

Target Category	Specific Molecular Target	Bioactivity	Reference
Antioxidant systems	ROS/RNS, SOD, GPX, Nrf2	scavenge free radicals and enhance endogenous antioxidant capacity	Yin et al. (2021) [10]
Inflammatory signaling pathways	NF-κB, MAPK, COX-2, IL-6 and iNOS	inhibit the release of inflammatory factors and reduce tissue inflammation	Wu et al., 2024 [11], Feng et al. (2018) [12]
Apoptosis/proliferation	Bcl-2, p53 and cyclin D1	regulate cell survival and inhibit abnormal proliferation	Faraone et al. (2020) [13]
Metabolic regulation/insulin signaling pathway	PPARs, AMPK, IRS/PI3K/Akt	improve glucose and lipid metabolism and enhance insulin sensitivity	Inoue et al. (2012) [14], Lewis et al. (2022) [15]
Mitochondrial protection	mPTP and mitochondrial membrane protein	protect mitochondrial membrane and inhibit the opening of mPTP	Baburina et al. (2019) [16]

AMPK: AMP-activated protein kinase; COX: cyclooxygenase; SOD: superoxide dismutase; GPX: glutathione peroxidase; IL-6: interleukin-6; iNOS: inducible nitric oxide synthase; ROS/RNS: reactive oxygen/nitrogen species; MAPK: mitogen-activated protein kinase; mPTP: mitochondrial permeability transition pore; PPARs: peroxisome proliferator-activated receptors.

**Table 4:** Anti-tumor effects of astaxanthin: evidence from animal studies.

Mechanism	Study Population/Model	Key Findings	Reference
Anti-tumor effect	Prostate cancer DU145 cell	Suppression of tumor cell proliferation and metastasis by inhibiting STAT3 expression	Sun et al. (2020) [54]
	PC-3 prostate cancer xenograft mouse model cells	Validated inhibitory effects on tumor growth	Ni et al. (2017) [55]
	Mouse skin papillomas	Antioxidant properties and capacity to scavenge peroxynitrite	Maoka et al. (2012) [56]
	SW480 cell and colorectal cancer mouse model	Curtails tumor cell proliferation and migration in colorectal cancer by regulating MAPK and NF-κB signaling pathways	Zhang et al. (2024) [57]
	C666-1 cell	Inhibits proliferation, migration, and invasion in nasopharyngeal carcinoma by blocking PI3K/AKT and NF-κB pathways via miR-29a-3p	Xu et al. (2024) [58]
	F344 rats	Inhibits the proliferation and migration of esophageal cancer cells by upregulating PPARγ expression	Cui et al. (2022) [59]
	U251MG cell	Hormetic effect in glioblastoma multiforme, where low concentrations promote cell proliferation, while high concentrations induce apoptosis	Tsuji et al. (2020) [60]

**Table 6:** Anti-diabetes effects of astaxanthin: evidence from animal studies.

Mechanism	Study Population/Model	Key Findings	Reference
Antioxidant Properties	Pancreatic β-cell	Protecting pancreatic β-cells, and enhancing insulin secretion	Sakayanathan et al. (2024) [102]
Anti-inflammation effect	Fat- and high-sucrose-diet-induced insulin-resistant mouse model	Strengthening endogenous antioxidant system, and mitigating oxidative damage	Liu et al. (2020) [103]
	Diabetic mice model	Decreasing GFAP-positive cells in the brain, downregulating the cleaved caspase-3, IL-6, and IL-1β, and upregulating CBS in the frontal cortex	Ying et al. (2015) [104]

## Abbreviations

The following abbreviations are used in this manuscript:

AD	Alzheimer’s disease
AFLD	alcoholic fatty liver disease
ALD	alcoholic liver disease
AMPK	AMP-activated protein kinase
APGS	astaxanthin polyethylene glycol succinate
AST	astaxanthin
AUC	area under the curve
bw	body weight
COPD	chronic obstructive pulmonary disease
COX	cyclooxygenase
CVD	cardiovascular diseases
EMT	epithelial–mesenchymal transition
FGF21	fibroblast growth factor 21
GPX	glutathione peroxidase
HDL	high-density lipoprotein cholesterol
HUVEC	human umbilical vein endothelial cell
IL-6	interleukin-6
iNOS	inducible nitric oxide synthase
LDL	low-density lipoprotein cholesterol
LPS	lipopolysaccharides
MAPK	mitogen-activated protein kinase
MASLD	metabolic dysfunction-associated steatotic liver disease
MCP-1	monocyte chemoattractant protein-1
mPTP	mitochondrial permeability transition pore
NO	nitric oxide
PD	Parkinson’s disease
PEG	polyethylene glycol
PGC-1α	peroxisome proliferator-activated receptor gamma coactivator 1α
PLGA	poly lactic-co-glycolic acid
PON1	paraoxonase 1
PPAR	peroxisome proliferator-activated receptors
ROS	reactive oxygen species
RNS	reactive nitrogen species
SOD	superoxide dismutase
SHR	spontaneously hypertensive rat
STING	Stimulator of Interferon Genes
TC	total cholesterol
TNF-α	tumor necrosis factor-alpha
VE-cadherin	vascular endothelial cadherin
